# p53 Transactivation and the Impact of Mutations, Cofactors and Small Molecules Using a Simplified Yeast-Based Screening System

**DOI:** 10.1371/journal.pone.0020643

**Published:** 2011-06-02

**Authors:** Virginia Andreotti, Yari Ciribilli, Paola Monti, Alessandra Bisio, Mattia Lion, Jennifer Jordan, Gilberto Fronza, Paola Menichini, Michael A. Resnick, Alberto Inga

**Affiliations:** 1 Unit of Molecular Mutagenesis, National Institute for Cancer Research, IST, Genoa, Italy; 2 Laboratory of Transcriptional Networks, Centre for Integrative Biology, CIBIO, Trento, Italy; 3 Chromosome Stability Group, Laboratory of Molecular Genetics, National Institute of Environmental Health Sciences, Durham, North Carolina, United States of America; University of Medicine and Dentistry of New Jersey, United States of America

## Abstract

**Background:**

The p53 tumor suppressor, which is altered in most cancers, is a sequence-specific transcription factor that is able to modulate the expression of many target genes and influence a variety of cellular pathways. Inactivation of the p53 pathway in cancer frequently occurs through the expression of mutant p53 protein. In tumors that retain wild type p53, the pathway can be altered by upstream modulators, particularly the p53 negative regulators MDM2 and MDM4.

**Methodology/Principal Findings:**

Given the many factors that might influence p53 function, including expression levels, mutations, cofactor proteins and small molecules, we expanded our previously described yeast-based system to provide the opportunity for efficient investigation of their individual and combined impacts in a miniaturized format. The system integrates i) variable expression of p53 proteins under the finely tunable *GAL1,10* promoter, ii) single copy, chromosomally located p53-responsive and control luminescence reporters, iii) enhanced chemical uptake using modified ABC-transporters, iv) small-volume formats for treatment and dual-luciferase assays, and v) opportunities to co-express p53 with other cofactor proteins. This robust system can distinguish different levels of expression of WT and mutant p53 as well as interactions with MDM2 or 53BP1.

**Conclusions/Significance:**

We found that the small molecules Nutlin and RITA could both relieve the MDM2-dependent inhibition of WT p53 transactivation function, while only RITA could impact p53/53BP1 functional interactions. PRIMA-1 was ineffective in modifying the transactivation capacity of WT p53 and missense p53 mutations. This dual-luciferase assay can, therefore, provide a high-throughput assessment tool for investigating a matrix of factors that can influence the p53 network, including the effectiveness of newly developed small molecules, on WT and tumor-associated p53 mutants as well as interacting proteins.

## Introduction

The sequence-specific transcription factor p53 is a key tumor suppressor protein that can coordinate the expression of a large number of target genes involved in different cellular responses to stress conditions including cell cycle arrest, programmed cell death and DNA repair [1], [2]. More recently, a role of p53 in a diverse spectrum of cellular pathways has been established, including angiogenesis, autophagy, as well as carbon and lipid metabolism [3], [4], [5]. p53 activity is finely tuned by a large number of signaling pathways which respond to alterations in cellular homeostasis or the microenvironment and result in the modulation of p53 protein levels, the potential for protein:protein interactions and DNA binding affinity/specificity. Modulation of the p53 network mainly occurs via post-translational modifications of the p53 protein itself [6]. The critical importance of p53 in tumor suppression in humans is exemplified by the high frequency of human cancers showing alterations in the p53 pathway, including p53 mutations [7].

Many studies in a variety of cell lines and *in vivo* animal models have provided striking evidence that the reconstitution of p53 activity can lead to tumor cell death as well as to the regression of established tumors [8], [9], [10], [11], [12]. Over the past 15 years such results have spurred a number of studies aimed at developing the means for restoring wild type p53 function in cells including viral delivery of p53 cDNAs and the rational design of small molecules or peptides that can stimulate p53 functions or reactivate tumor-associated mutant p53 proteins [13], [14], [15]. In tumors that retain wild type p53, the regulated pathway is frequently, if not always, impaired by other genetic events that result in higher expression and activity of the critical negative p53 regulator MDM2 or, to a lesser extent MDM4 and other modulators of p53 protein localization and activity [16], [17], [18], [19]. The critical roles of MDM2 and MDM4 as negative modulators of p53, which have been elegantly established using knock-out models [20], [21], as well as the over-expression of these proteins in several cancer types [17], [22], [23] raised expectations on the therapeutic potential of restoring p53 functions by MDM2/4 in tumors. However, the identification of chemicals that could disrupt protein:protein interactions or protein:DNA interactions involving p53 has proven challenging [24].

Small molecules that can inhibit the interaction between MDM2 and p53 can result in increased p53 protein levels and lead to p53-dependent growth suppression and apoptosis in different cell-based as well as *in vivo* models [25], [26], [27]. For example, Nutlin and the MI-43 compounds target the binding pocket for p53 in the MDM2 protein. RITA, which was identified in a cell-based screening assay, binds p53 and also inhibits the p53:MDM2 interaction [25]. Structural studies have identified similarities as well as shape differences between the p53-binding pockets in MDM2 and MDM4 [28], supporting the selectivity of Nutlin in p53:MDM2 interactions [29].

To investigate the impact of small molecules on p53 transactivation potential or on the functional interaction between p53 and cofactors, we have developed a highly defined dual-luciferase functional assay in the budding yeast *Saccharomyces cerevisiae*. This greatly expands our previous system designed to address functions of p53 mutants and target response elements by varying the level of p53 [30], [31]. The assay exploits the variable expression of p53 proteins and utilizes the *Firefly* and *Renilla* luminescent reporters integrated as single copies at different chromosomal loci in haploid strains or at the same chromosomal location in diploid strains, *i.e.,* heteroalleles. While a common minimal promoter controls low-level constitutive expression of both reporters, p53-dependent expression of the *Firefly* reporter is attained through a specific p53 response element (RE) placed upstream of the minimal promoter [32], [33].

The sensitivity and robustness of the assay system was investigated with various protocols for induction of wild type and mutant p53 protein as well as coincident measurement of the two luciferases. This was followed by an examination of the ability of the dual assay system to discern the functional interaction of wild type and mutant p53 when co-expressed with MDM2 or 53BP1 and the effects of RITA and Nutlin. Our results establish that the functional interactions as well as the impact of the small molecules were distinct and depend on the nature of the p53 mutants. The responsiveness to these chemicals did not extend to PRIMA-1 which has been reported to restore apoptotic activity of specific tumor-associated p53 missense mutants in engineered cancer cells [34], [35], [36]. We propose that our dual-luciferase yeast assay can be applied to the study of small molecules in order to investigate their differential impact on a large number of tumor-associated p53 mutations as well as partial inactivation of wild type p53 [37]. Furthermore, unlike other p53 screening systems, our genetically well-defined, cell-based assay can be applied to high-throughput screening (HTS) of chemicals toward a matrix of factors that can influence the p53 network including p53 protein levels, p53 mutations, nature of the p53 REs, and level of p53-interacting proteins.

## Materials and Methods

### Drugs, plasmids and media

RITA was purchased from Cayman Chemical (Cayman Europe, Tallinn, Estonia), Nutlin from Alexis Biochemicals (Enzo Life Sciences, Milan, Italy), and PRIMA-1 was obtained from Inalco (Inalco, Milan, Italy). Stock solutions of the compounds were prepared at the concentration of 10 mM; RITA and Nutlin were prepared in DMSO while PRIMA-1 was dissolved in water. Working dilutions were freshly prepared in yeast culture media immediately before treatment.

pTSG-hp53 was used to express human wild type or mutant p53 protein under the control of the *GAL1* inducible promoter. The plasmid is based on the centromeric pRS314 vector and contains the *TRP1* selection marker. Plasmids pRB254 and pRB759 were used to express MDM2 and 53BP1, respectively. These *HIS3*-marked plasmids were obtained from Rainer Brachmann (Irvine University, CA, USA) and contain full-length MDM2 or a 53BP1 fragment lacking the first 970 amino acids, that are constitutively expressed under the PGK1 and ADH1 promoter, respectively. Given that our luciferase reporter strains could not support *HIS3*-based plasmid selection due to a cryptic mutation in the histidine biosynthesis pathway, to conduct experiments with the co-expression of p53 and MDM2 or 53BP1 we constructed a diploid yeast reporter strain by mating our strain (whose construction is described below) yLFM-PUMA, RFM-M2, Δ*pdr5* [*Matα his-, leu2, trp1, ura3, ade2::cyc1-LUC, pdr5::cyc1-REN*] with the BY4704 strain (*Mat* ***a*** *ade2::hisG; Dhis3-200; leu2-Δ0; lys2-Δ0; met15-Δ0; trp1-Δ63,* where “Δ0” indicates complete removal of the ORF sequence). The resulting diploid is heterozygous for Δ*pdr5*. Plasmids were transformed into yeast cells using the standard LiAc protocol. Transformants were picked and purified on selective plates containing glucose as carbon source. To conduct the luciferase assays while exploiting variable induction of p53 proteins, yeast cells were cultured in liquid media containing 2% raffinose (Sigma-Aldrich, Milan, Italy) as carbon source or 2% raffinose supplemented with different amount of galactose (Sigma-Aldrich) as inducer of the *GAL1* promoter (as indicated in the Results section and Figure Legends) following the protocol developed previously [31], [32], [33], [38]. All media components were obtained from BD-Bioscience (BD-Biosciences Italy, Milan, Italy) or Sigma-Aldrich. 5-Fluoroorotic Acid was purchased from Toronto Research Chemicals Inc. (North York, Ontario, Canada). The integrative plasmid pdr1DBD-repressor (sin3) was a generous gift of Dr. John Nitiss (St. Jude Children's Hospital, TN, USA) and was used to disrupt the regulator of the ABC transporter system *PDR1* gene by replacing it with a fusion construct whereby the *PDR1* DNA binding domain is fused with the *SIN3* transcriptional repression domain [39].

### Development of dual-luciferase yeast reporter strains

The *Renilla* luciferase open reading frame (ORF) was amplified from the pRL-SV40 vector (Promega, Milan, Italy) and integrated at the *ADE2* locus using the *delitto perfetto* approach [40] starting from the available y-FM-cyc1-ICORE- strain [32]. This strain contains the targeting module, consisting of the I-SceI recognition site and GAL1-I-SceI expression cassette, that provides for generation of a single, site-specific double strand break by the homing endonuclease I-SceI. The targeting module also contains a *URA3* and a *KANMX4* marker, respectively, for counter-selection on plates containing 5-fluoro-orotic acid and forward selection for G418 resistance [41]. The *ICORE* was integrated by exploiting homologous recombination downstream of the minimal *CYC1* promoter and in place of the *ADE2* ORF in the previously developed yAFM strains [38]. ICORE replacement with the *Renilla* ORF resulted in the yRFM (R  =  *Renilla*) strain which was further modified by introducing the ICORE cassette upstream of the minimal *CYC1* promoter. The resulting yRFM-ICORE strain was then used to develop desired p53 RE insertions upstream of the *CYC1* promoter by targeting the ICORE site with oligonucleotides containing the chosen RE sequences, as previously described [38]. To develop dual-luciferase yeast reporter strains two approaches were followed. To construct an isogenic diploid reporter, the yRFM strain, in which *Renilla* is expressed at basal levels, was transformed by pGAL-HO plasmid [42], [43] and cultured in galactose to induce expression of the HO endonuclease in order to induce mating type switching. The yRFM, *Mat* ***a*** derivatives were identified by crosses with mating type testers, purified and then used in a cross with the yLFM-PUMA p53 reporter strain. The resulting diploid strain is isogenic, but hetero-allelic at the *ADE2* locus, in that one chromosome contains the *Firefly* luciferase, while the other contains *Renilla*. The diploid version of the assay was used for the experiments investigating the impact of MDM2 or 53BP1 on p53 transactivation potential.

A haploid dual-luciferase reporter strain was also developed placing the *CYC1-Renilla* construct at the *PDR5* locus. First, we targeted the *PDR5* gene that codes for a p-glycoprotein whose disruption results in increased sensitivity to a broad spectrum of chemicals [44]. To this aim the *PDR5* gene was modified by PCR-mediated integration of the ICORE disruption cassette starting from the yLFM-PUMA strain. The resulting yLFM-PUMA *pdr5::ICORE* strain was then further modified by replacing the *ICORE* cassette with a PCR product obtained by amplifying the *Renilla* reporter cDNA starting from the yRFM strain and using PCR primers containing tails of homology for the *ICORE* integration flanking sites at the *PDR5* locus. Alternatively, the *ICORE* cassette was removed from the *PDR5* locus using a short oligonucleotide to simply recycle the cassette and leave a complete deletion of the targeted gene. Sequences of all primers for targeting and colony PCR analysis are available upon request.

### Small volume dual luciferase assays in yeast

Yeast transformants were selected on plates selective for the presence of the *p53/MDM2/53BP1* expression vectors. Overnight cultures (1 ml) were grown in glucose liquid medium to keep p53 expression repressed. The cultures were then washed in selective medium containing 2% raffinose as carbon source and diluted to OD_600nm_ ∼0.1 in media containing 2% raffinose and a desired amount of galactose (see Results section) for the induction of the *GAL1* promoter that drives p53 expression. 100 µl of cell suspensions were placed in 96-well plates. When needed the desired concentration of the small molecules RITA, Nutlin and PRIMA-1 were added to the cell suspension in the 96-well format. The 96-well plate was then incubated for 16 hrs at 30°C under moderate (150 rpm) orbital shaking. Immediately prior to the luciferase assays, cultures were resuspended and 10 µl were transferred to a white 384-well plate. OD_600_ was directly measured in the 96-well plate. For the luciferase assay, 10 µl of PLB buffer 2X (Passive Lysis Buffer, Promega, Milan, Italy) were added to the 10 µl cell cultures, and the 384-well plate was placed on a thermomixer and incubated for 15 min at room temperature with the shaker set at 500 rpm. 10 µl of *Firefly* luciferase Bright Glo substrate (Promega, Milan, Italy) were then added to the cell suspension and light units were measured in a plate reader (Mithras LB940 plate reader -Berthold Technologies, Milan, Italy or Infinite M-200, Tecan, Milan, Italy). For the dual-luciferase assay, 5 µl of the *Firefly* luciferase substrate (Luciferase Assay Reagent, LARII, Promega) followed by 5 µl of the Stop&Glow buffer were used instead of the Bright Glo, (Promega) to measure Renilla activity.

### Larger volume luciferase assay in yeast

The results obtained with the newly developed small volume luciferase assay were compared to those obtained with an intermediate protocol that utilized 1 ml liquid cultures to induce p53 expression. Luciferase activity was determined without the laborious extraction of soluble proteins by mechanical lysis and centrifugation. To this aim, 0.5 ml of the cultures were collected by centrifugation after the 16-hour growth in the desired p53-inducing conditions. Cells were suspended in 0.5 ml of 1x PLB (or CCLR) lysis buffer and incubated for 15 min. at room temperature. 10 µl of cell suspensions were then transferred to a white 96-well plate and 50 µl of Bright Glo reagent were added for the luciferase assay. 100 µl of the cell suspension were also transferred to a transparent 96-well plate to measure the OD_600nm_ that was used as normalizing factor. The dual luciferase assay was developed similarly, except for the use of 10 µl of the *Firefly* substrate and 10 µl Stop&Glow® *Renilla* substrate.

### Protein extraction and luciferase assay

The results obtained with the newly developed small volume luciferase assay were also compared with the previously developed protocol that relies on 1 to 2 ml liquid cultures of yeast transformants and soluble protein extraction [31], [45]. Briefly, purified transformants with the desired p53 expression plasmids were cultured to induce p53 expression for 16 hrs in 2 ml of synthetic selective medium. Cells were then collected by centrifugation, washed in sterile water and suspended in 100 µl of GLO lysis buffer (Promega, Milan Italy) and an equal volume of pre-chilled glass-beads (∼0.5 mm, Sigma-Aldrich) was added. Protein lysates were obtained from mechanical lysis of the cells obtained using a vortex mixer. Protein extracts were cleared by centrifugation (15 min at ∼16000 g at 4°C) and quantified using the BCA Protein Assay (Pierce Biotechnology, Milan, Italy). Luciferase activity was measured using a multilable Mithras LB940 plate reader (Berthold technologies, Milan, Italy) or Infinite M-200 plate reader (Tecan) using 10 µl of extracts and 50 µl of the Bright-Glo assay reagent (Promega).

### Western Blot

Yeast transformants were grown overnight in selective galactose-containing medium and an equivalent amount of cells, based on the culture absorbance measurement (OD_600nm_), were collected the day after in 1.5 ml tubes by centrifugation (1 min ×14000 g). Cells were washed once with 1 ml of sterile water and harvested again by centrifugation. Pellets were then resuspended in 300 µl of lysis buffer (0.025 M Tris-HCl pH 6.8, 0.015 M NaCl, 10% glycerol, additioned with 0.01 M PMSF and 1x complete protease inhibitor cocktail (Roche, Milan, Italy). One volume of acid-washed glass beads (0.4–0.5 mm diameter, Sigma, Milan, Italy) was added to the cell suspension and lysis was obtained by 6 cycles of 30 sec. vortex at high setting, each followed by 30 sec. on ice. Soluble proteins were then obtained after centrifugation at 4°C for 10 min. at maximun speed. Supernants were transferred and proteins quantified using the BCATM method (Pierce, Thermo Scientific Milan, Italy). Protein extracts were boiled at 95°C for 5 min., resolved with SDS-Page on 7.5% BisTris Acrylamide gels using a Biorad MiniProtean III apparatus (Bio-Rad, Milan, Italy) and transferred to Nitrocellulose or PVDF membranes using the semidry iBlot system (Life Technologies, Milan, Italy). After blotting the quality as well as the equal loading and transfer of protein blots was determined by Ponceau S staining. The membranes were probed with monoclonal or polyclonal antibodies specific for p53 (pAb1801 and DO-1, Santa Cruz Biotechnology) MDM2 (SMP14, Santa Cruz Biotechnology, D.B.A. Italia, Milan, Italy) and actin (I-19-R, Santa Cruz Biotechnology). The relative Molecular mass (*M*_r_) of the immunoreactive bands was determined using molecular weight markers (Fermentas, Milan, Italy). After washing, blots were incubated with the appropriate IgG- horseradish peroxidase conjugated secondary antibody (Santa Cruz Biotechnology), and immune complexes were visualized with ECL plus reagent (GE Healthcare) using a Molecular Imager ChemiDoc XRS+ system (Bio-Rad). Band intensities were quantified using the Image-Lab software (Bio-Rad).

## Results

### Development of a small-volume, dual-luciferase assay to study p53 transactivation potential

In previous studies [30], [31], [32], [33], [38], we reported several modifications to the original yeast-based *ADE2* color (red/white) p53 functional assay [46]. The p53 gene was placed under the control of the finely-tuned inducible *GAL1,10* promoter (“rheostatable”) to address transcriptional issues that are dependent upon protein levels. This system revealed subtle differences in p53 function at many target sequences and identified mutants with enhanced or altered transactivation capacity including change-of-spectrum mutants [47]. The *ADE2* reporter was replaced with the more quantitative *Firefly* luciferase and the system incorporated a convenient *in vivo* mutagenesis system based on oligonucleotides [40] that enabled us to easily create isogenic yeast reporter strains differing only in the p53 RE target sequence driving the luciferase reporter [38]. The resulting system provided opportunities to address the transactivation potential of p53REs, functional SNPs in p53 REs and noncanonical REs [30], [31], [32], [33].

**Figure 1:**
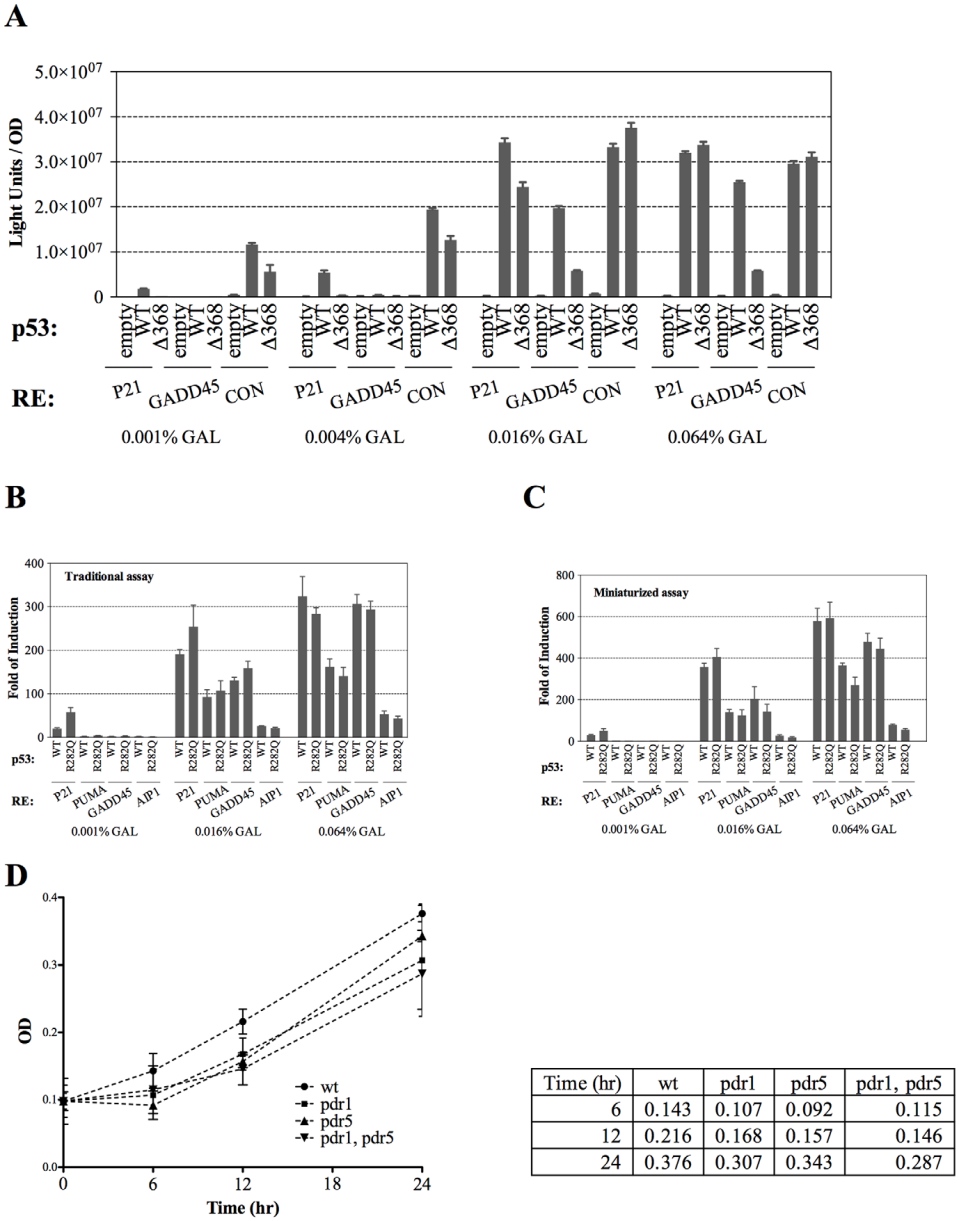
Generation of a small volume format for p53 functional assays. (A) Relative transactivation capacity of WT p53 and a carboxy terminal deletion measured in permeabilized cell cultures and normalized to optical density OD. p53 proteins were induced at different levels by varying the amount of galactose, as indicated. Three different p53 response elements (REs) that differed in relative transactivation capacity from very strong (CON, an optimized consensus sequence), to strong (P21, corresponding to the p21-5′ site) and to moderate (GADD45). OD of the cultures was used as normalizing factor. Presented are the average measurements and standard deviations of three biological replicates. (B, C) Small-volume yeast cultures can determine p53 transactivation capacityRelative transactivation capacity of WT and the R282Q p53 have been compared towards four different REs obtained with the traditional assay based on 2ml liquid cultures in individual tubes (B) and with the permeabilized assay format based on 100 µl cultures prepared directly in 96-well plates (C). p53 proteins were induced at different levels by varying the amount of galactose, as indicated. A strong (P21), two moderate (PUMA, GADD45) and a weak RE (AIP1) were compared. Cells collected from the two different culture protocols were used for the measurement of luciferase activity as described in the Materials and Methods section. Presented are the average fold-induction of luciferase by p53 proteins relative to the activity obtained with an empty vector; included is the standard deviations of three replicates. (D) Impact of genetic modifications of the ABC-transporter systems on yeast growth. Overnight liquid cultures in synthetic medium containing glucose were washed and resuspended in fresh medium containing raffinose (2%) as the carbon source and low levels of galactose (0.0032%) (time zero) to induce p53 protein expression. Cultures were diluted to ∼0.1 OD_600nm_, as measured by a plate reader. OD was measured at the 6, 12, 24hr time intervals. Error bars plot the standard deviations of three biological replicates. The average absorbances are also presented to the right of the graph.

While very informative, the requirement for 1 to 2 ml cultures per experimental condition and soluble protein extraction to quantify *Firefly* luciferase activity limited the experimental opportunities. Thus, we sought to develop a miniaturized system that did not require protein extraction. As described in the following, we found that cells in growth phase as well as stationary of both the haploid and diploid strains we developed could be permeabilized for uptake of luciferase substrate if resuspended in Passive Lysis Buffer (PLB) or Cell Culture Lysis Reagent (CCLR) from Promega (Milan, Italy) without leading to the appearance of soluble protein in the solution. There was a time-dependent loss of viability in PLB buffer (survival was ∼10% after 1 hr incubation at room temperature). Cells were incubated for 10 min in the PLB prior to the addition of the *Firefly* luciferase substrate. Since the permeabilized cells retained structural integrity, the optical densities (OD_600nm_) of cell suspensions could be used for normalization. The assay provides robust measurement of p53-dependent transactivation, as shown in Figure 1A. The transactivation potential of wild type (WT) p53 and the Δ368 deletion mutant lacking the regulatory domain in the p53 carboxy terminus (C-ter) were determined using three reporter strains and four galactose concentrations to modulate p53 expression. The results are in agreement with our previous analysis of the same p53 proteins and REs using luciferase measurements following protein extraction [31].

We also examined the robustness of the system for detecting luciferase activity within small culture volumes (100 µl) using 96-well plates and sampling 10 µl aliquots. In these experiments both WT and the partially functional R282Q mutant were expressed at variable levels under the inducible *GAL1* promoter (Figure 1B and C) as well as under the constitutive *ADH1* promoter (Supporting Information S1). Relative transactivation potential was measured from four p53 REs: the strongly responsive P21-5′, the moderate GADD45 and PUMA and the weaker AIP1 [38]. Again, the results were comparable to those obtained with the traditional protein extraction and luciferase protocol (compare Figure 1B with Figure 1C) supporting the use of luminescent reporters, permeabilized cells, and small volumes to assess p53 transcriptional functions as well as providing a high-throughput format.

### Genetic modification of reporter strains to improve drug accumulation

To make our assay more suitable to test different kind of molecules, we modified the ABC transporter genes to increase the accumulation of small molecules. Specifically, we took advantage of a disruption cassette for the *PDR1* (pleiotropic drug resistance) gene, a regulator of the ABC-transporter system, that replaces the WT gene with a chimeric construct in which the PDR1 DNA binding domain is fused to a transcriptional repressor domain. This chimeric gene provides dominant enhanced sensitivity to a variety of chemicals [39] in yeast. We also disrupted the p-glycoprotein gene *PDR5,* resulting in increased sensitivity to a broad spectrum of chemicals [44], [48], [49]. Growth of the ABC mutants was examined in liquid cultures under the same conditions used for the luciferase protocol described above (see Materials and Methods). As shown in Figure 1D, the growth rates appeared comparable to WT in raffinose and galactose-containing medium after an initial delay following transfer from glucose medium (Figure 1D). The same results were observed both in rich and synthetic, glucose-containing medium (Supporting Information S1 and data not shown). For all the galactose concentrations used in this study (up to 0.064%) we did not detect an impact of p53 expression on growth parameters of the yeast cultures nor a distinct impact of the genetic modifications targeting the ABC transporter system (not shown). To examine the impact of these genetic modifications on drug accumulation in our strain background, we evaluated the toxicity of cycloheximide [39] (Supporting Information S1). Results confirmed that both *PDR1* and *PDR5* disruption rendered the cells more sensitive to the drug. The *pdr5* mutant was the most sensitive although, surprisingly, the double mutant *pdr1, pdr5* exhibited a slightly reduced sensitivity compared to *pdr5*. Although the specific impact of the PDR1 or PDR5 deletions could be dependent on the nature of the small molecule tested [48], based on the observed relative sensitivity in this work we focused on the *pdr5* mutant to develop the modifications of the yeast-based assay.

### Dual-luciferase system to study p53-dependent transactivation

**Figure 2:**
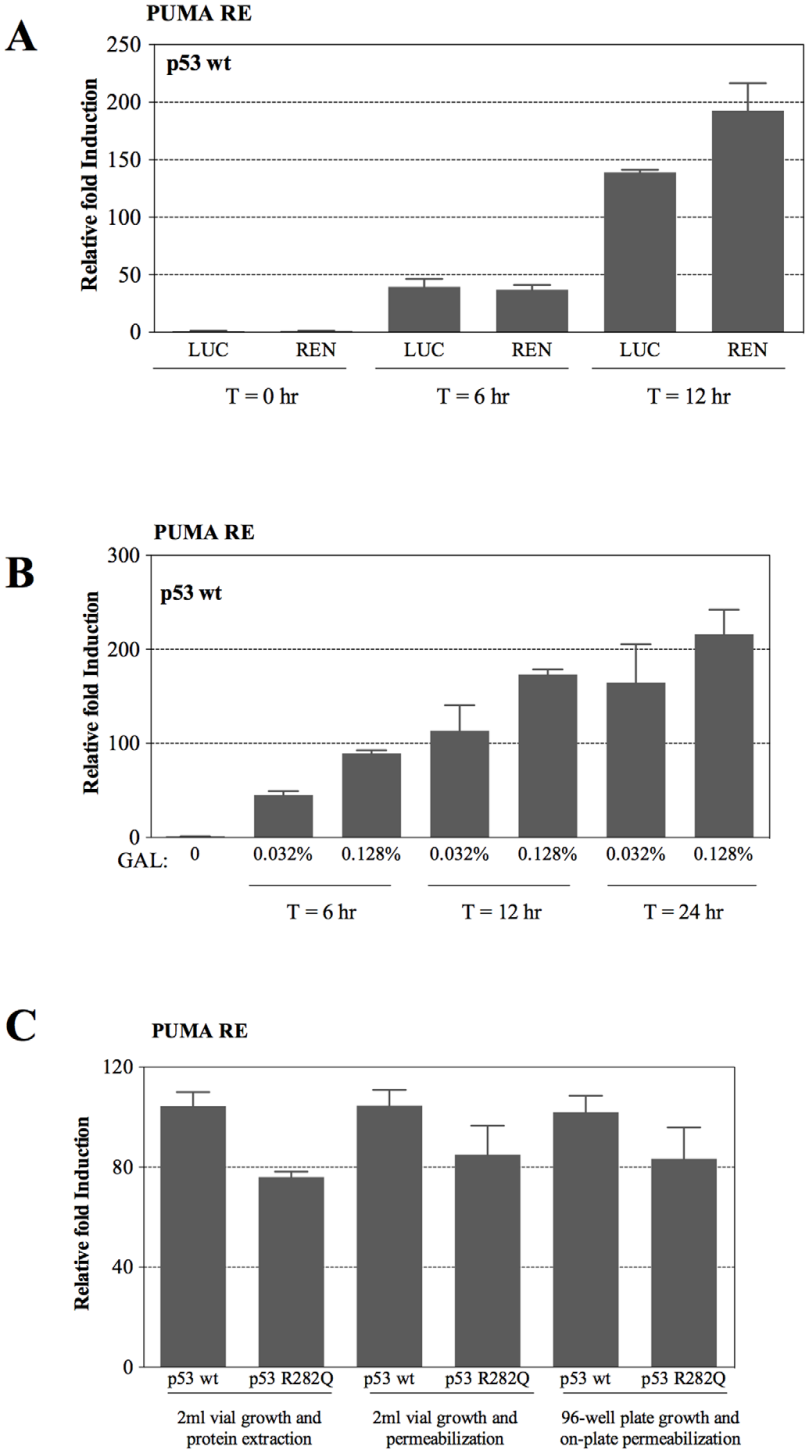
Either *Firefly* or *Renilla* luciferase can function as p53-dependent reporters. (A) The ability of *Firefly* and *Renilla* cDNAs to serve as reporters for p53 transactivation was examined by placing them downstream from the moderate p53 RE derived from the PUMA promoter in isogenic strains. The values indicate the fold induction measured over an empty vector. Presented are average and standard deviations of three replicates relative to optical density of the cultures measured at different times (T in hrs) after switching cultures to galactose-containing medium. (B) Dual luciferase reporter assay with a strain expressing WT p53 and containing the *Firefly* luciferase as p53 reporter gene and the *Renilla* luciferase as constitutive reporter. Presented are the average and standard error of the *Firefly* luciferase activities normalized for *Renilla* and compared to empty vector at various time points after shifting 100 µl yeast cultures to galactose-containing media in the 96-well plate format. (C) Comparison of relative induction using measurement of protein from 2 ml cultures vs direct permeabilization of cells in a 384 well format following transfer from a 96-well growth plate, as described in the text and the Materials and Methods section. Relative transactivation capacities of WT p53 and the R282Q mutant in the “2 ml vial”experimental set-ups were measured using either protein extraction or permeabilization. Experiments were conducted using 0.032% galactose inducer, unless specified otherwise. Error bars plot the standard error of four biological replicates.

The system was further modified to include a *Renilla reniformis* cDNA luminescent reporter that could be used for internal normalization rather than relying on cell density. We established that *Renilla* activity can also be measured in cell suspensions prepared in PLB or CCLR lysis buffers by comparing p53-dependent transactivation potential in a pair of strains containing the *Firefly* or the *Renilla* reporters cloned downstream of the moderate p53 RE derived from the human PUMA target gene (the OD provided a normalizing parameter), as shown in Figure 2A. To develop the *Renilla* reporter as an internal standard, the *Renilla* cDNA was placed downstream from the minimal *CYC1* promoter, previously used for the *Firefly* luciferase [38] without the introduction of a p53 RE. This *CYC1-Renilla* minimal promoter-reporter cassette, which provides constitutive basal (low-level) expression of the control luciferase, was cloned at the *PDR5* locus (*pdr5:REN*) in the yLFM-PUMA reporter strain. The p53 responsiveness of this dual-luciferase system is depicted in Figure 2B. Luciferase activities could be detected using only 10 µl of cell suspension and 5 µl of standard luciferase substrate and were comparable to those obtained using our previous approaches that involved lysis by glass beads and larger volumes [31], [33].

To directly compare the different approaches for detecting p53 transactivation, the WT p53 and the R282Q mutant were tested in the following manner: i) 2 ml cultures of cells were lysed with glass beads; ii) 500 µl of the 2 ml cultures were transferred into 1.5 ml eppendorf tubes and permeabilized with 100 µl of 1x lysis buffer; and iii) 100 µl cultures were incubated in 96-well plates of which 10 µl were transferred into 384-well plates and permeabilized by an equal volume of 2x PLB lysis buffer. As shown in Figure 2C, the miniaturized dual-luciferase assay provides a sensitive and robust system for addressing p53 dependent transactivation in a format that is amenable to high-throughput screens.

### Functional interactions between p53 and MDM2 or 53BP1 using the yeast-based dual luciferase assay

A goal in the development of the dual luciferase system was to obtain a suitable assay to address interactions of p53 with factors that determine its stability and to identify chemicals that could modify those interactions. Specifically, we addressed the functional interaction between p53 and MDM2 and the impact of small molecules targeting this interaction. MDM2 is a critical inhibitor of p53 functions that can bind p53 in the amino terminal (N-ter) region and lead to p53 protein degradation via its E3 ubiquitin-ligase activity in human cells [50]. For these experiments we generated diploid reporter strains that could select for the MDM2 expression vector (see Materials and Methods). The diploid cells were heterozygous for the PDR5 deletion. Consistent with a previous report [51], we found that in yeast MDM2 co-expression resulted in a reduction of p53-dependent transactivation. Initially, we explored the impact of MDM2 on the ability of increasing amounts of p53 protein to transactivate the *ADE2* red/white reporter from an upstream p53 RE [30], [38]. The MDM2 cDNA was expressed constitutively under the moderate *PGK1* promoter. Reduction of p53-dependent transactivation by MDM2 was observed only at very low levels of p53 expression (Supporting Information S1, raffinose only vs raffinose + galactose plates) and was affected by amino acid changes in the p53 N-ter mimicking post-translational modifications. The MDM2 inhibition of p53 activity in this semi-quantitative assay was dependent on the p53 RE examined and was observed only with the highest p53 affinity REs, p21-5′ and CON, being suitable for the *ADE2* reporter assay.

**Figure 3:**
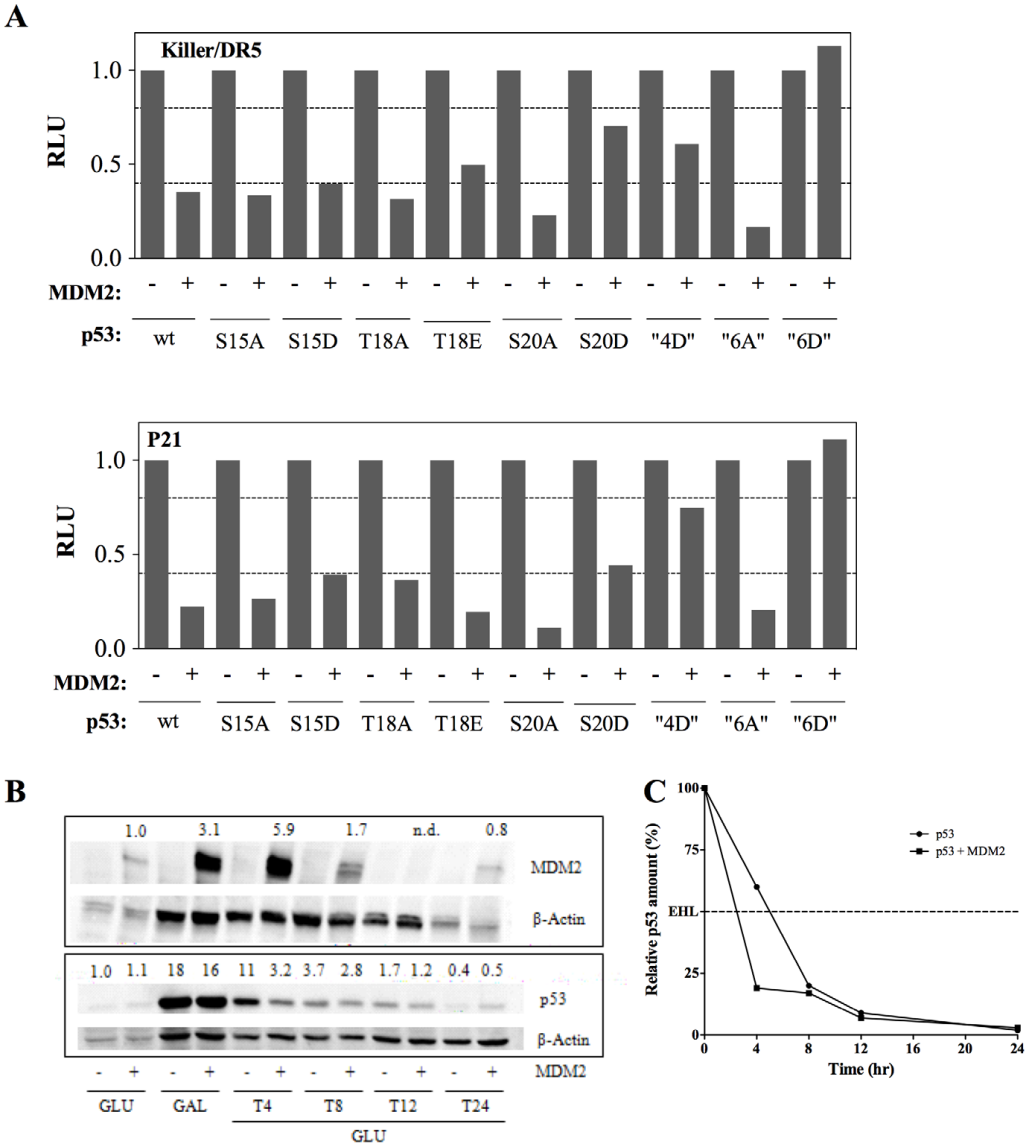
MDM2 co-expression reduces WT and mutant p53-dependent transactivation and can impact p53 protein level and stability. (A) The functional interaction between p53 and MDM2 was examined using two different reporter strains, as indicated. Transformants were cultured in 0.012% galactose to achieve low expression of p53 for 16 hours. MDM2 is expressed under the constitutive PGK1 promoter. Besides WT p53, several mutants at Ser/Thr in the p53 N-ter were tested. The activity of each p53 mutant was set to one to better focus on the relative impact of MDM2 co-expression on p53 transactivation capacity. The relative transactivation potential of the various p53 mutants is presented in Supporting Information S1; 4D refers to a quadrupole mutant with S15D, T18E, S20D, S33D changes in p53 . 6A indicates a multiple mutant with alanine changes at S15, T18, S20, S33, S37, S46. 6D indicates a multiple mutant with aspartic acid changes at S15, S20, S33, S37, S46 and a glutamic acid change at T18. Presented are the average fold-inductions by p53 proteins compared to empty vector and normalized using the *Renilla* control luciferase. These assays were conducted with diploid strains that were obtained by crossing the indicated yLFM- p53 reporter strains with the BY4704 strain (see Materials and Methods section) using the permeabilized format. (B) Western blot analyses of p53 and MDM2 protein levels. O/N cultures in synthetic glucose medium (GLU) were washed and shifted to medium containing raffinose and 0.012% galactose (GAL) to achieve low expression of p53. The p53 was expressed under the inducible *GAL1* promoter while MDM2 was expressed at constitutive levels from a moderate *PGK1* promoter. After 16 hrs of growth in galactose-containing medium, cells were washed and transferred to glucose medium to repress the *GAL1* promoter. Samples were collected at the indicated time points to prepare protein extracts for western blot. 100 µg (MDM2 and actin, top panel) and 20 µg (p53 and actin, lower panel) of extract was loaded in each lane. The DO-1, SMP14 and I-19-R antibodies (Santa Cruz) were used for the immunodetection of p53, MDM2 and actin, respectively.Actin levels were used as a normalization factor to estimate relative MDM2 and p53 amounts. Consistent with a previous study [55], we observed that MDM2 expression under the PGK1 promoter was affected by the culture state and was particularly reduced when cell approached the stationary phase (O/N in glucose; T8 and T12 time points; at T12 cells were diluted for the additional 12 hr time point). The relative changes in MDM2 and p53 protein amounts compared to the level observed in glucose cultures are indicated above the immunoblot. (C) Quantification of p53 expression relative to the amount observed after 16 hrs in 0.012% galactose, normalized to actin levels. A 10% reduction in steady-state p53 protein amount due to the co-expression of MDM2 was observed in the galactose-induced cultures. To better visualize the impact of MDM2 on the estimated p53 half life (EHL) the relative amount of p53 observed after 16 hrs in galactose was set to 100%, both for extracts of cells expressing only p53 or p53 + MDM2.

The impact of MDM2 on p53 WT and mutants was subsequently evaluated using a luciferase-based assay. Specifically, serine/threonine residues in the N-terminal domain were mutated to mimic phosphorylation events in mammalian cells or to prevent phosphorylation; these residues are modified as part of the signaling pathways that activate p53 by influencing protein:protein interactions including that with MDM2 [52], [53], [54]. As shown in Figure 3A, we confirmed that MDM2 could inhibit p53-dependent transactivation from different p53 REs. The impact of the mutations was in part dependent on the nature of the p53 RE driving *Firefly* luciferase expression. In particular the T18E and S20D p53 mutants were less sensitive to MDM2-dependent inhibition of transcription at a moderate RE (Killer/DR5) than with a strong RE (p21-5′). On the contrary, transactivation at either RE by p53 mutants mimicking constitutive phosphorylation (referred to as “4D” and “6D” in the figure) was largely insensitive to co-expressed MDM2. The transactivation potential of those N-ter p53 mutants when expressed alone was comparable to WT p53 with the exception of the multiple mutant 6A, where the concomitant change of Ser 15, 20, 33, 37, 46 as well as of threonine 18 into alanine resulted in approximately three-fold higher activity (Supporting Information S1).

The impact of MDM2 co-expression on WT p53 protein levels was also assessed using western blot analysis (Figure 3B & C). p53 protein levels were determined from cells grown in glucose (steady-state) or from cells grown in galactose for 16 hrs to induce p53 and then transferred to glucose media to repress the transcription of the p53 cDNA to estimate the p53 protein half life in yeast. p53 protein amounts were quantified relative to β-actin loading control. A 10% reduction in steady-state p53 protein amount due to the co-expression of MDM2 was observed in the galactose-induced cultures (Figure 3B lanes 3 & 4). Furthermore, MDM2 appeared to reduce p53 half life in yeast, based on relative quantitation of the immunoblot at the various time points after the transfer of the cells to glucose medium. p53 half life was estimated to be ∼2.5 hours in cells that express MDM2 and 5 hours when MDM2 was not expressed (Figure 3C). MDM2 protein levels also appeared to vary during the experiment, in relation to the growth phase of the cultures. A previous study reported that the PGK1 promoter that controls MDM2 cDNA expression in the vector we used, could be severely repressed in stationary phase cells, while remaining largely unaffected by changes of carbon sources in the medium [55]. It is important to note that all the luciferase assays in our work were conducted in cultures grown for 16 hrs in galactose-containing medium, when cells are still in a late-logarithmic culture phase. A previous study in yeast where MDM2 and p53 were co-expressed under a GAL promoter reported a similar impact of MDM2 on p53 protein half life [56].

Overall, these results strongly suggest that the functional interaction between p53 and MDM2 is at least in part dependent on the same amino acids in the p53 N-ter domain as in mammalian cells but does not lead to a strong reduction in p53 protein stability. Thus, the impact of MDM2 in yeast is likely due to a competing effect for p53 binding to components of the transcription machinery, as suggested previously [51].

**Figure 4:**
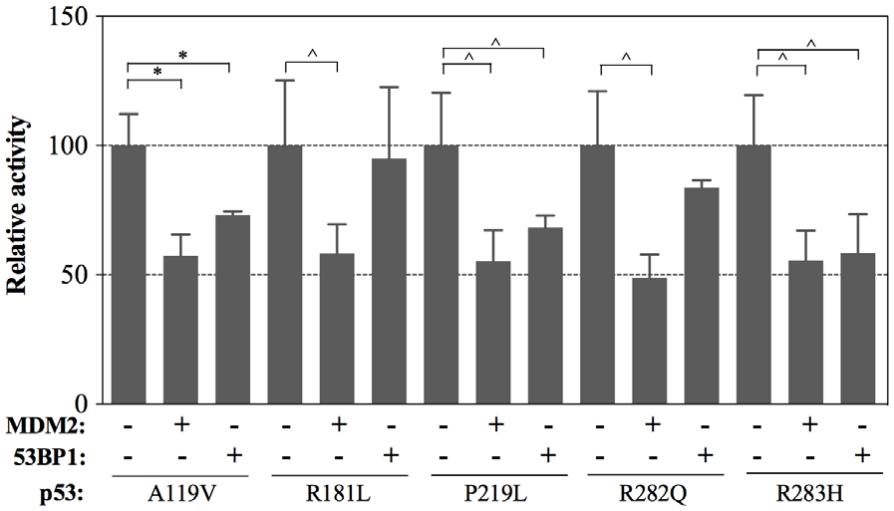
Functional interactions between partial function p53 mutants and MDM2 or 53BP1. Mutant p53 expression was under the control of the *GAL1* promoter while MDM2 or 53BP1 (a clone containing a N-ter deletion of the first 970 amino acids) were expressed at constitutive levels under the *PGK1* and *ADH1* promoters, respectively. p53 expression was induced for 16 hrs in medium containing 0.012% galactose. Presented are results describing the impact of MDM2 or 53BP1 on transactivation of various p53 mutants that are capable of partial transactivation toward the PUMA RE. To better visualize the impact of MDM2 and 53BP1, the activity of each p53 mutant alone is set to 100%. The relative light units of the various mutants in this experiment were WT p53, 2.1×10^5^; A119V, 1.3×10^5^; R181L, 0.86×10^5^; P219L, 0.87×10^5^; R282Q, 0.79×10^5^; R283H, 0.53×10^5^. Significant differences in activity relative to p53 alone are shown (*: p<0.01; ∧: p<0.05, Student's t-test).

**Figure 5:**
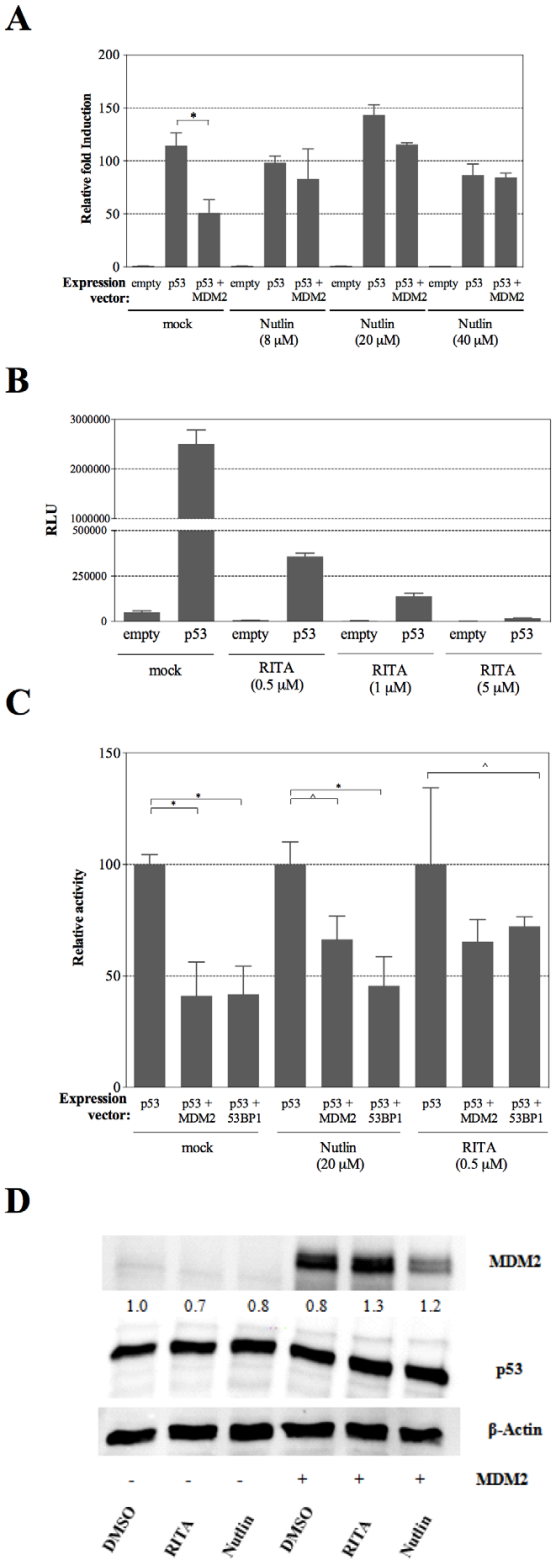
Functional interactions between wild type p53 and MDM2 or 53BP1 and the impact of Nutlin and RITA. WT p53 was expressed at low-level achieved by culturing cells in medium containing 0.012% galactose for 16 hrs in the 96-well plate format. MDM2 was expressed from the moderate PGK1 promoter. (A) The impact of MDM2 on p53-dependent transactivation was examined in the presence of different concentrations of Nutlin added to the medium at the time of the switch to galactose-containing medium using a reporter strain containing the moderate PUMA p53 RE. The average transactivation relative to the basal level of reporter activity measured in cells that do not express p53 and standard deviations of three biological repeats are presented. Significant differences in activity relative to p53 alone are shown (*: p<0.01, Student's t-test). (B) *Firefly* luciferase activities normalized using the control luciferase *Renilla* are presented for empty vector and wild type p53 in the presence of different amounts of RITA. (C) Nutlin and RITA impact on the functional interactions between p53 and MDM2 or 53BP1. Nutlin (20 µM) or RITA (0.5 µM) were added at the time of switching cultures to galactose-containing medium. The luciferase activity by wild type p53 alone, normalized using the *Renilla* control luciferase, is set at 100%. Both MDM2 and 53BP1 co-expression reduced p53-dependent transactivation. Nutlin partially relieved the functional impact of MDM2, but not that of 53BP1. RITA partially relieved p53 from the inhibition by both MDM2 and 53BP1. Significant differences are shown (*: p<0.01; ∧: p<0.05, Student's t-test). (D) MDM2 and p53 immunoblot in mock-, RITA- and Nutlin-treated yeast cells. Proteins were prepared from cells grown in medium containing 0.012% galactose for 16 hrs and treated with DMSO solvent control 0.5 µM RITA or 20 µM Nutlin. 25 µg were loaded to detect p53 and 100 µg of protein extracts were loaded to probe for MDM2. Actin was used as a loading control.

We extended the study of p53 interactors in the dual-luciferase system to 53BP1 using a yeast-expression vector containing a 53BP1 clone with N-ter deletion of the first 970 amino acids [51]. 53BP1 was identified in a 2-hybrid screen by its ability to bind the DNA binding domain (DBD) of p53 through the BRCT domains present in the C terminal region (C-ter) of 53BP1 [57]. While 53BP1 was shown to act as a positive cofactor for p53 function in human cells [58], its co-expression with p53 (WT or mutant) in our yeast-based assay led to a reduction in p53-dependent transactivation (Figure 4 and 5C), consistent with a previous study [51]. This result indicates that 53BP1 might compete with p53 for sequence-specific binding to DNA. Unlike the general inhibition by MDM2, the impact of 53BP1 differed towards specific partial-function p53 missense mutants, consistent with the p53:53BP1 physical interaction. For example, transactivation by the R181L and R282Q mutant proteins were slightly or not affected by 53BP1, while the transactivation by A119V, P219L and R283H was reduced by co-expression of 53BP1 (Figure 4).

### The small molecules Nutlin and RITA can reduce the inhibitory effects of MDM2 and 53BP1

Using the dual luciferase system we then investigated the impact of two well-known small molecules than can affect p53, namely Nutlin and RITA. The former can disrupt the interaction between p53 and MDM2 by binding to MDM2 while RITA can interfere on the same interaction by targeting p53, possibly leading to conformational changes [59]. As summarized in Supporting Information S1, we did not observe a significant impact on yeast growth under the conditions used for the WT strain or for the ABC-transporter mutants, although the mutants experienced a delay in growth following the shift in media (also described in Figure 1). While Nutlin had little impact on transactivation by p53 alone it counteracted the negative impact of MDM2 (Figure 5A).

Treatment with RITA led to a severe reduction in p53-dependent Firefly luciferase activity (Figure 5B). However, also the basal luciferase activity was affected by RITA, indicating that the effect might not be related to p53. A negative impact of RITA on *Firefly* luciferase activity was previously reported in mammalian cells [60]. However, RITA had no impact on the basal activity of the *Renilla* luciferase. We, therefore, constructed a dual luciferase reporter strain in which the *Renilla* luciferase was placed under p53 transcriptional control. In this strain, treatment with RITA had no effect on p53-induced transactivation detected by the *Renilla* luciferase (Supporting Information S1). After taking into account the impact of RITA on basal *Firefly* luciferase activity, we were able to show that RITA could partially relieve the inhibition of p53-dependent transactivation by MDM2 (Figure 5C). The impact of Nutlin and RITA on the p53/53BP1 functional interaction was also examined. Treatment with Nutlin did not modify the 53BP1-dependent inhibition of p53-dependent transactivation (Figure 5C). However, the inhibition was partially relieved by RITA. Western blot analysis confirmed that MDM2 co-expression had little impact on p53 protein levels after culturing cells for 16 hrs in 0.012% galactose. Interestingly, treatment with Nutlin but not RITA appeared to reduce MDM2 expression/stability (Figure 5D).

### PRIMA-1 exhibited an apparent lack of impact on p53 mutants

The dual luciferase system was investigated for its responsiveness to PRIMA-1, a small molecule identified in a mammalian cell-based screen for chemicals that could induce apoptosis in a mutant p53-dependent manner [35]. PRIMA-1 restored sequence-specific DNA-binding and transcriptional transactivation to mutant p53 *in vitro*, possibly through altering mutant p53 conformation or folding stability [61] although the precise mechanism remains to be determined. To examine the impact of PRIMA-1, we chose a panel of p53 mutations that differ in their relative transactivation capacity in the yeast-based assay. Four loss-of-function mutants were tested, including the two cancer hotspot mutants R175H and R273H that were shown to be responsive to PRIMA in human cells [35]. We also examined 5 partial function p53 mutations since they could register negative and positive impacts of small molecules. The transactivation potential of the p53 mutants ranged from 50 to 80% of the WT protein in the reporter strain containing the PUMA p53 RE under moderate expression from the *GAL1* promoter. As described in the supplementary material, we were unable to detect any effect of PRIMA-1 on transcription by WT or mutant p53 in WT (not shown) or in *pdr5* mutant cells (Supporting Information S1).

## Discussion

In this study we have greatly expanded the features of our previously described yeast strains for assessing p53 and p53 RE function in order to develop a system that is both more efficient and miniaturized. The system provides for rapid assessment of p53 transactivation potential as well as the impact of p53 mutations, cofactors and small molecules. In particular, it integrates variable expression of p53 proteins under the finely tunable *GAL1* promoter, single copy luminescence reporters that are chromosomally located with opportunities to co-express p53 alleles along with chosen cofactor proteins coded from selectable low copy number plasmids. Furthermore, the assay is based on a small-volume format for p53 expression, treatment with chemicals, and quantification of the reporter expression and is compatible with high-throughput screening.

### Interactions with p53-cofactor proteins

Specifically, we have established that the new, dual-luciferase based protocol can assess p53-dependent transactivation and the impact of single amino acid changes in the p53 DBD. We found that co-expression of MDM2 can lead to reduced p53 transactivation at low levels of p53 protein expression. p53 mutants at the DBD that have partial transactivation function were also inhibited by MDM2, whereas mutations introduced into the p53 N-ter domain that mimic phosphorylation events could relieve p53 from the MDM2-dependent inhibition. Those same amino acid changes did not alter significantly the transactivation potential of the p53 protein, when expressed alone. This suggests that the assay can be used to reveal ectopic p53:MDM2 physical interactions that are likely to occur at the p53 N-ter region, similar to the endogenous interaction in higher eukaryote cells. The assay also revealed a modest impact of MDM2 on p53 protein stability in yeast. Although MDM2 was recently found to bind p53 at the DNA binding domain (DBD) and at the C-ter [62], the primary site of interaction occurs at the transactivation domain (TAD) in the p53 N-ter region [63].

We also examined the impact on p53 transaction of another important p53 cofactor, the protein 53BP1. The BRCT domains present in the 53BP1 C-ter are required for the interaction with p53 as well as with other important proteins such as BRCA1 [57]. The physical and functional interactions between p53 and 53BP1 in the context of DNA damage response appears to be complex. Following DNA damage, 53BP1 can localize to nuclear foci in mammalian cells, is rapidly phosphorylated in an ATM-dependent manner [64], and is essential for DNA double strand break repair [65]. Furthermore, 53BP1 appears to be an important mediator of the induction of senescence and cell death pathways elicited by BRCA1 deficiency in mice [66]. A crystal structure of p53 DBD bound to the human 53BP1 BRCT domains led to the identification of amino acids in the p53 DBD involved in such interaction [67]. More recently, the Tudor domain of 53BP1 was shown to interact with p53 proteins dimethylated in the p53 C-ter region at lysine 382 [68]. The generation of p53 dimethylated at Lys382 promotes the accumulation of p53 protein that occurs upon DNA damage but this accumulation is dependent on 53BP1 [69]. These results suggest that the positive coactivator function of 53BP1 towards p53 in mammalian cells [58] may be related to its positive impact on p53 protein amount. Possibly, 53BP1 reduces the interaction between p53 and MDM2 (G. Selivanova, unpublished results).

The co-expression of 53BP1 with p53 leads to a reduction in p53-dependent transactivation, similar to previously reported findings in yeast [51]. Unlike MDM2, the impact of 53BP1 was lost or greatly reduced with specific partial function p53 mutants in the DBD. For example the p53 R181L mutant was not sensitive to 53BP1. Structural studies showed that p53 R181 formed both a hydrogen bond and stacking interactions with 53BP1 residues in the BRCT domains [67]. The reduced interaction between the p53 mutant R282Q and 53BP1 could not be linked to the reported physical interaction between the two proteins.

Overall, our results indicate that the dual-luciferase yeast-based assay can be used to study the interaction between p53 and cofactor proteins. While the functional interaction appears dependent on conserved physical interactions, the outcomes of the co-expression on p53-dependent transactivation in the yeast assay does not always reflect expectations from mammalian cells, although such discrepancies can be reasonably explained and related to the defined nature of the assay, as proposed above for the impact of 53BP1.

### Impact of small molecules

Having established that the yeast-based assay can reveal a functional interaction between p53 and its cofactors MDM2 or 53BP1, we explored the impact of small molecules targeting those interactions using Nutlin and RITA. Nutlin had been isolated as a small molecule that interacts with the p53-binding pocket in MDM2, resulting in accumulation of p53 protein and possibly inhibition of MDM2 activity towards other of its targets [10], [59], [70]. Treatment with Nutlin led to p53 accumulation in a variety of cancer cell lines, without significant induction of p53 post-translational modifications, and resulted mainly in cell cycle arrest, although apoptosis was also detected. The compound showed p53-dependent growth suppression in *in vivo* experiments without much evidence for toxicity in nude mice. The small molecule RITA (*r*eactivation of p53 and *i*nduction of *t*umor cell *a*poptosis) was obtained in a cell-based assay screening for induction of WT p53-dependent apoptosis [25]. Its mechanism of action appears to be at least in part related to a direct interaction with the p53 protein and inhibition of the p53-MDM2 binding. Differently from Nutlin, which directly affects the binding of MDM2 to the amino-terminal region of p53, RITA was reported to bind the p53 N-ter region and indirectly affect the functional interaction with MDM2 [59], [70]. RITA could induce p53-dependent apoptosis in a variety of tumor cell lines [25].

Our results establish that treatment of yeast cells with the small molecules Nutlin or RITA could partially relieve WT p53 from the MDM2-dependent inhibition, similar to what is observed in mammalian cells. Furthermore, while Nutlin treatment had no impact on the functional interaction between p53 and 53BP1, RITA was also able to target the p53/53BP1. Combined with the observation that 53BP1 appeared to interact with p53 mutants in a manner that is mutant-specific, our results suggest that the yeast-based assay could be used to screen a large panel of tumor-associated p53 mutations for differential impact of these chemicals on p53 functional interaction with cofactors.

Attempts to modify WT or mutant p53 function by PRIMA-1 were unsuccessful. PRIMA-1 was reported to restore the sequence-specific DNA-binding and transcriptional transactivation of some p53 mutants *in vitro* and to suppress tumor-cell growth in mice by inducing apoptosis (Bykov *et al.*, Nat Med. 2002). Interestingly, PRIMA-1 inhibited the growth of cell lines derived from various human tumor types in a mutant p53-dependent manner [71]. The precise mechanism of action of this compound is not clear; moreover its selectivity for mutant p53 remains to be fully established and may also be related to indirect effects on p53 folding and nuclear localization. For example PRIMA-1 induced the expression of heat shock protein 90 (Hsp90) in breast cancer cells, restored the p53-Hsp90 interaction and enhanced the translocation of the p53-Hsp90 complex to the nucleus [72]. Recently the ability of PRIMA-1 to induce nucleolar localization and degradation of mutant p53 protein has been demonstrated [73], suggesting the existence of a complex mode of action, likely cell-type specific, that can be independent from the restoration of transactivation functions to mutant p53. Indeed, PRIMA-1 fails to stimulate the DNA binding potential of isolated mutant p53 DBD *in vitro* [59]. The apparent lack of effect of PRIMA-1 in our assay might be due to poor uptake, even in the *pdr5* mutant, or modification of the chemical in yeast. It has been shown that PRIMA-1 is converted to compounds that forms adducts with thiols on mutant p53 and such p53 protein modifications can trigger apoptosis [61]. It might well be that these activating modifications are impaired in yeast.

### Overview

Cell-based functional assays are expected to be useful tools for identifying molecules targeting mutant p53 or impacting on the interaction between p53 and cofactors. They can provide unbiased screening opportunities for leads that act beyond steric hindrance of protein:protein interactions including allosteric modifiers of protein folding or stability. Allosteric modulators could be combined potentially with rationally designed drugs to increase potency or overcome single agent resistance *in vivo* [74]. In this regard initial studies suggest that the combination of Nutlin and RITA might provide additional stimulation of p53-induced responses, consistent with the different broad transcriptional responses induced by the two compounds when given as single agents [75]. However, off-target effects that impact the biological endpoints being measured, such as the induction of apoptosis, can hamper identification of mechanisms of action of molecules scoring positively in cell-based screening assays. This potentially limiting feature is especially relevant in the case of proteins like p53, whose functions are wired into many cell-signaling pathways. Furthermore, the tremendous variability in tumor-associated p53 mutations and in expression levels of distinct p53-interacting proteins and p53 splice and promoter variants as well as p53-related proteins p63 and p73 could significantly affect the outcome of small molecule treatments. The yeast-based assay described here has the advantage of generally being free of p53 biological consequences. Alternatively, assays have been developed that exploit the impact of moderate/high levels of p53 expression on the growth of yeast [76], [77]. This type of assay provides the opportunity to score the effect of cofactors or small molecules that may also act on p53 transcriptional-independent functions. However, the exact mechanisms of p53-mediated growth retardation in yeast are not well-defined. The growth retardation could be, in part, dependent on effects on transcriptional complexes, based on our previous identification of toxic p53 alleles in yeast that at low expression levels result in enhanced transactivation capacity and on the loss of the toxicity caused by second-site loss-of-function missense mutations in p53 ([47], [78].

The spectrum of missense p53 mutations associated with sporadic and familial cancer comprises more than 1200 distinct sporadic and ∼110 germline mutations (www.iarc.fr/P53/) [79]. Furthermore, biochemical, and functional assays have revealed that the degree of thermodynamic as well as folding instability caused by the mutations and their impact on sequence-specific transactivation function can vary greatly [30], [80], [81]. These differences could impact the activity of small molecule modifiers. Furthermore, the efficacy of allosteric modifiers could be significantly affected by the cellular/nuclear amounts of p53 mutant proteins or by the ratio between wild type p53 and specific negative cofactors, such as MDM2 or MDM4. Finally, the impact of small molecules could be, in part, influenced by the nature of the interaction between p53 and its many different cognate response elements located in the large number (hundreds) of human p53 target genes [82], [83], [84].

In summary, we propose that the miniaturized yeast dual luciferase system we developed provides a genetically well-defined, robust and cost-effective assay that can be used in parallel to mammalian cell-based assays to screen molecules or further evaluate leads that target p53 functions. A specific advantage of the assay is the potential for high-throughput assessment of a matrix of factors that include low and variable levels of p53 proteins, nature of the p53 response elements and specific, disease-associated p53 mutations. All these variables could impact the activity of small-molecule modifiers of p53 functions. Our assay system could be particularly relevant for further characterization of small molecules that may act as allosteric modifiers of p53 functions or p53-cofactor interactions.

## Supporting Information

Supporting Information S1 **1. Small-volume yeast functional assay with constitutive expression of p53 proteins.** Presented is the comparison of the relative transactivation capacity of wild type (WT) and the R282Q p53 towards four different response elements (REs) obtained with the traditional assay based on 2 ml liquid cultures in individual tubes (A, traditional assay) and with the permeabilized assay format based on 100 µl cultures prepared directly in 96-well plates (C, miniaturized assay). p53 proteins were expressed under the moderate, constitutive ADH1 promoter. Cells collected from the two different culture protocols were used for the measurement of luciferase activity as described in the Materials and Methods section. Presented are the average fold-induction of luciferase by p53 proteins relative to the activity obtained with an empty vector; included is the standard deviations of three replicates. In these experiments the light units per OD for WT p53 and the p21-5′ RE were 2.8×10^6^ for the 2 ml cultures and 2.5×10^7^ for the 100 µl cultures. **2. Impact of genetic modifications at the ABC transporter system on cell sensitivity to cycloheximide.** Based on the experiments described by Stepanov *et. al.* [39] we used cycloheximide treatment to evaluate whether the disruption of *PDR1* and replacement with the *PDR1*-repressor construct, the disruption of *PDR5*, or the combined modifications would result in enhanced toxicity in our reporter strain background. Cells from the indicated strains were resuspended in sterile water and transferred to a 96-well plate. Serial dilutions (1:5) were prepared and cells were transferred to plates containing synthetic medium (SD) with different concentrations of cycloheximide using a 48-pin replicator. A rich (YPDA) and an SD control plates were also spotted for comparison. Plates were incubated for two days at 30°C. **3. Phenotypic analysis of the impact of MDM2 on WT and mutant p53 transactivation.** The ADE2-based red/white assay was used to examine p53 dependent transactivation and the impact of MDM2. p53 was expressed at low levels under the *GAL1* promoter in media containing only raffinose (2%), or raffinose plus 0.002%, 0.004% or 0.016%, galactose. MDM2 was expressed from the constitutive *PGK1* promoter. p53 transactivation was examined from three REs upstream of ADE2-based p53 reporter strains as indicated. The optimized consensus (CON) and P21-5′ p53 RE yield levels of high transactivation while the NOXA RE is weaker [38]. In the *ADE2*-based p53 functional assays, cells grown on plates containing a low-amount of adenine (5 mg/L) result in small red colonies when p53 is not present or not transcriptionally active. p53-dependent expression of *ADE2* results in the appearance of colonies with a color ranging from light red to white, depending on the level of transactivation. To reveal the dependency of the phenotype on p53 expression levels, streaks are prepared on glucose plates containing high amount of adenine (200 mg/L) and the plates are incubated for two days at 30°C, resulting in the appearance of white colonies. These plates are then replica-plated to a stack of plates containing 2% raffinose plus various levels galactose along with the low-level of adenine. The replica plates are then incubated at 30°C for 2-3 days. Images of a section of the replicas are presented. For each image the upper section corresponds to colonies expressing p53 alone, while in the lower section the colonies also express MDM2. Various multiple mutants were tested, as indicated. **4. Relative transactivation capacity of p53 phosphorylation-site mutants.** The activity of the p53 mutants described in Figure 4 is presented as relative light units in two p53 reporter strains using the Killer/DR5 or the p21-5′ REs upstream of a luciferase reporter. Results were obtained with the traditional assay format and are normalized to amount of soluble proteins. 4D refers to a quadruple mutant with the S15D, T18E, S20D, S33D changes in p53. 6A indicates a multiple mutant with alanine changes at S15, T18, S20, S33, S37, S46. 6D indicates a multiple mutant with aspartic acid changes at S15, S20, S33, S37, S46 and a glutamic acid change at T18. **5. Impact of small molecules Nutlin and RITA on the growth of WT yeast reporter strains or the isogenic derivatives with modified chemical uptake.** Overnight cultures grown in synthetic glucose medium were washed and diluted to ∼0.1 OD_600nm_ as measured by a plate reader. (A) The WT strain was treated with 40 µM or 80 µM Nutlin (indicated as nutlin 1 and nutlin 2 respectively) and 1 µM or 2 µM RITA (indicated as rita1 and rita2). (B) The indicated mutant ABC-transporter strains were treated with1 µM RITA (or DMSO solvent control). OD was measured at the following times: 2, 4, 8, 12 and 24 hrs. Error bars correspond to the standard deviations of three biological replicates. **6. Negative impact of RITA on the** ***Firefly*** **luciferase but not the** ***Renilla*** **luciferase.** To confirm that the negative impact of RITA on the *Firefly* reporter was not dependent on modulation of p53 transactivation, an isogenic derivative strain was developed containing the PUMA p53 RE upstream of the *Renilla* luciferase. Wild-type p53-dependent transactivation was examined in cultures treated with DMSO control solvent or with 1 µM RITA. Presented are relative light units normalized to OD_600nm_ of the cultures. The error bars correspond to standard deviations for three biological repeats. **7. Apparent lack of PRIMA-1 effects on yeast growth or p53-dependent transactivation.** *(A) The small molecule PRIMA-1 does not affect yeast growth.* Overnight cultures grown in synthetic glucose medium were washed, diluted to ∼0.1 OD_600nm_, as measured by a plate reader, and treated with 200 µM PRIMA-1. Growth curves were compared for the wild type strain or the indicated ABC-transporter mutants. OD_600nm_was measured at the 2-, 4-, 8-, 12- and 24-hr time points. Presented are standard deviations for three biological repeats. *(B) The small molecule PRIMA-1 does not impact wild type p53 transactivation capacity.* Cells were grown in glucose-containing media to keep p53 expression repressed and transferred to galactose-containing media followed by the addition of PRIMA-1. Dual luciferase assays were conducted 16 hrs after the treatment. *Renilla* luciferase was used as normalization factor. There was no significant effect of PRIMA-1 on WT p53 transactivation. The same result was obtained with a diploid yeast strain, in which both the p53-dependent reporter (*Firefly*) and the control luciferase (*Renilla*) were placed at the *ADE2* chromosomal locus (*i.e.,* heteroalleles), thus removing potential chromatin effects on reporter expression. The diploid strain was obtained starting from two isogenic isolates of our yLFM strain background that differ for the mating type locus. Presented is the fold-induction of the *Firefly* reporter over the *Renilla* reporter relative to strains that do not express p53, as they contain an empty expression vector. *(C) The small molecule PRIMA-1 does not affect mutant p53 transactivation capacity.* Different p53 alleles were expressed at moderate levels using medium containing 0.128% galactose. PRIMA-1 (200 µM) was added to the cultures at the time of the switch to galactose-containing medium. Presented are the average fold-induction by p53 proteins compared to empty vector and normalized using the *Renilla* control luciferase. Presented are standard deviations for three biological repeats.
(DOC)
