# Supplementary figures and images for: p53 Transactivation and the Impact of Mutations, Cofactors and Small Molecules Using a Simplified Yeast-Based Screening System
Source: PLoS One. 2011 Jun 2;6(6):e20643. doi: 10.1371/journal.pone.0020643 (PMC3107237; doi:10.1371/journal.pone.0020643)

**Supporting Information S1**

**1)**


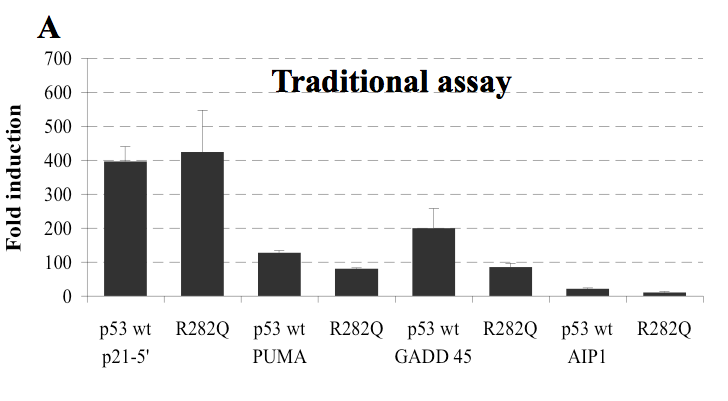


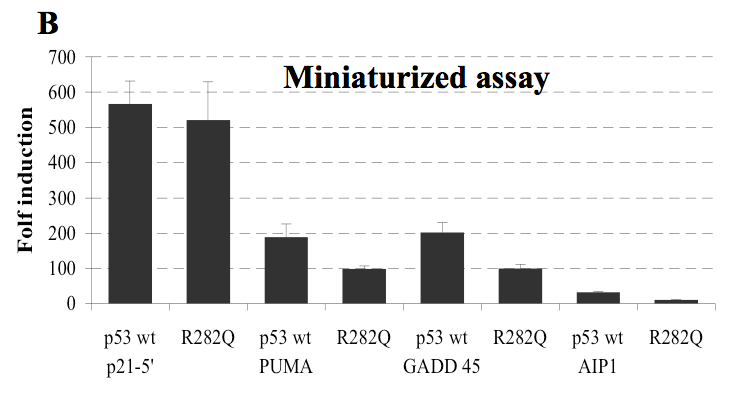


**2)**


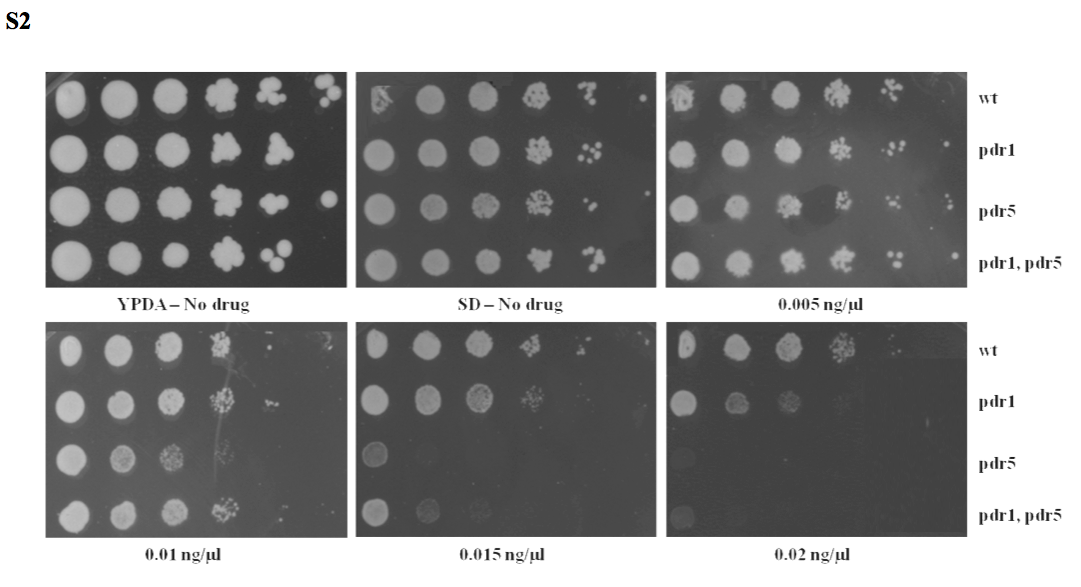


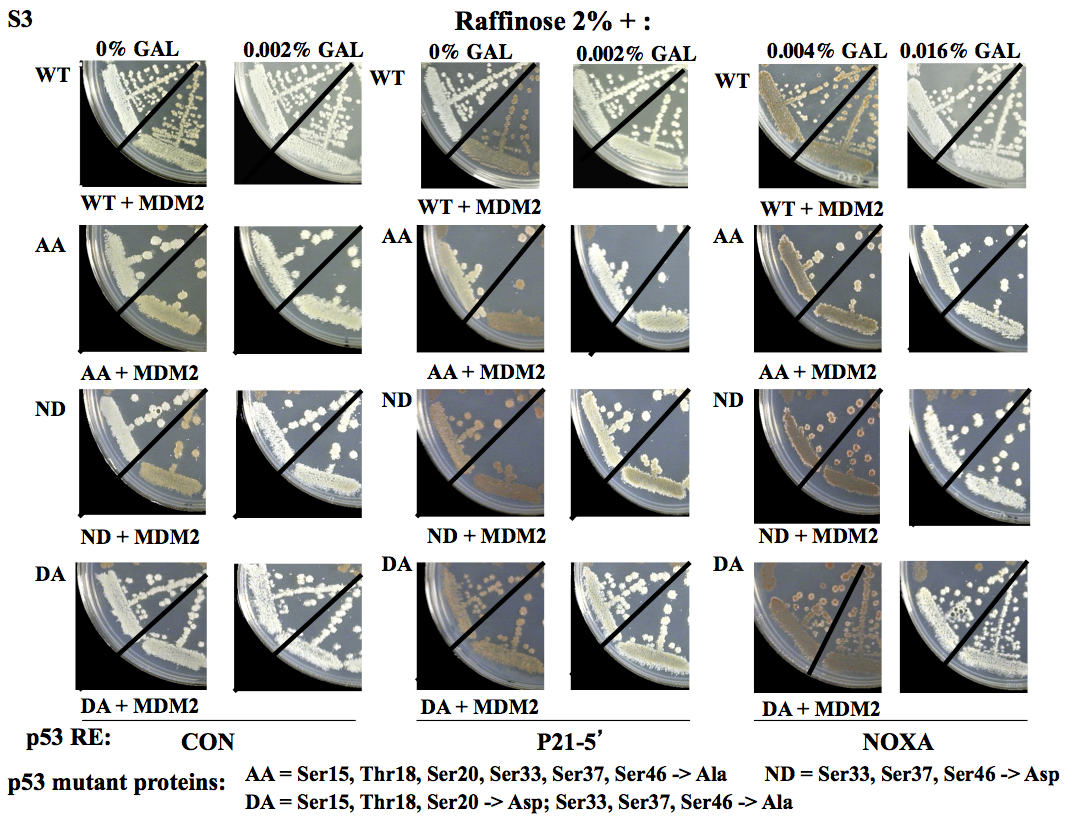


**4)**


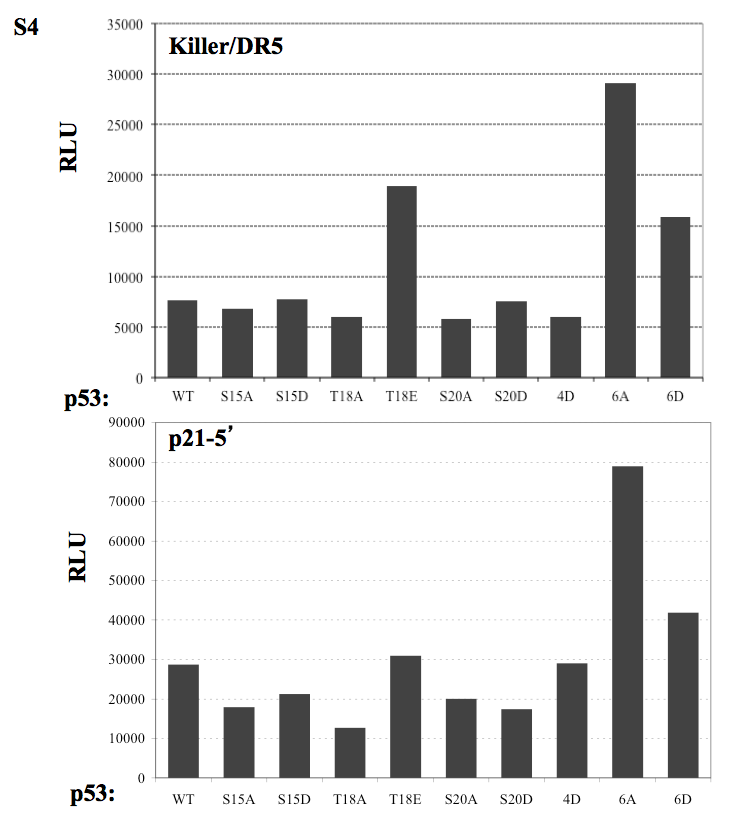


**5)**


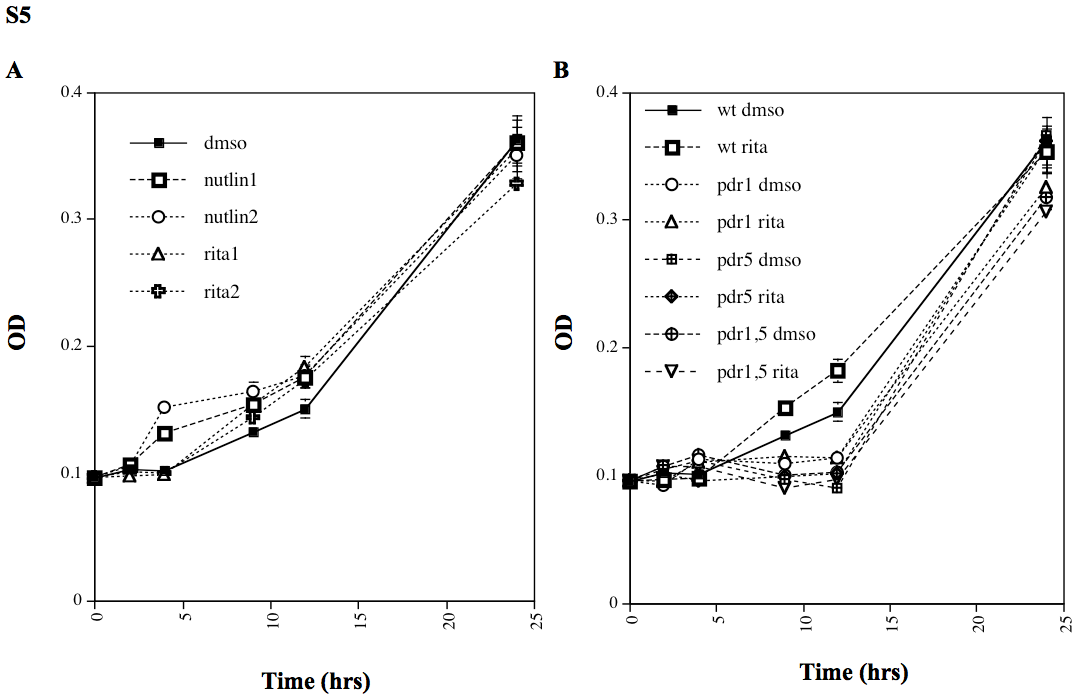


**6)**


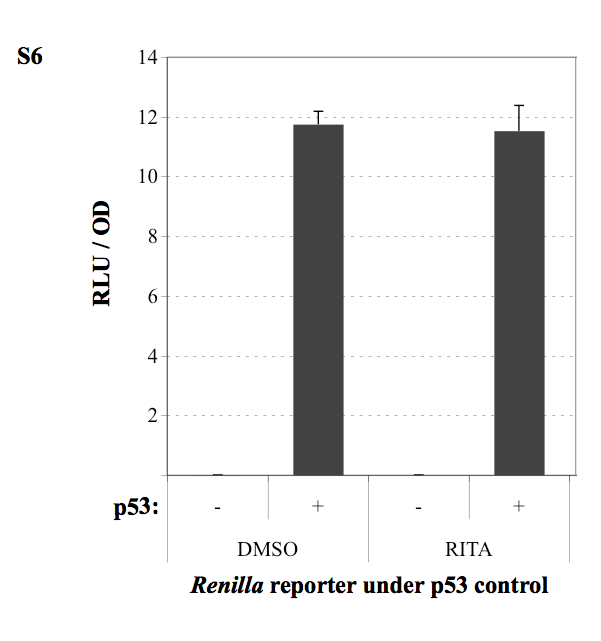


**7)**


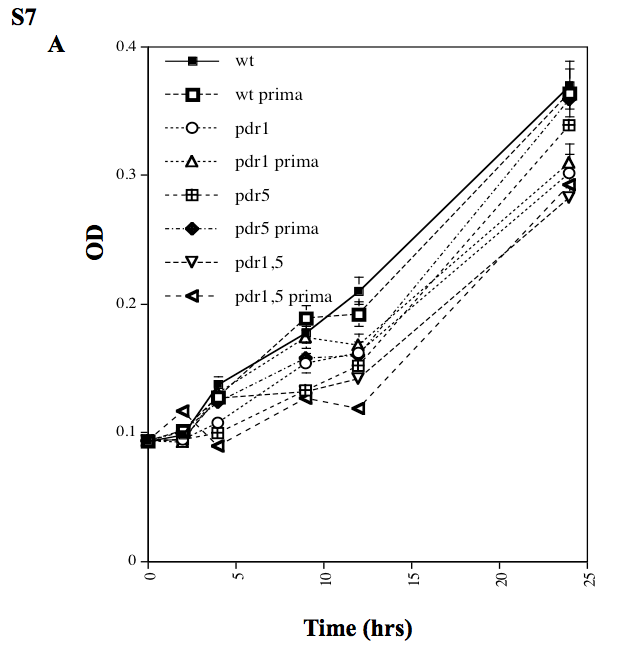


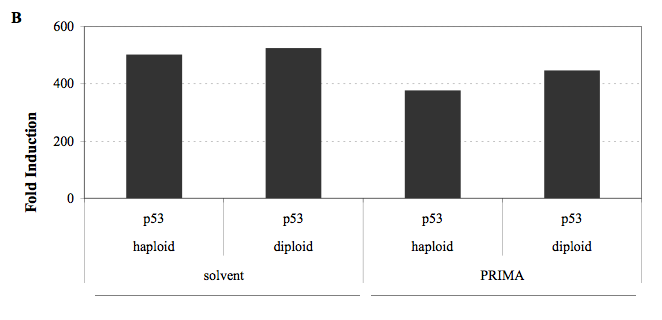


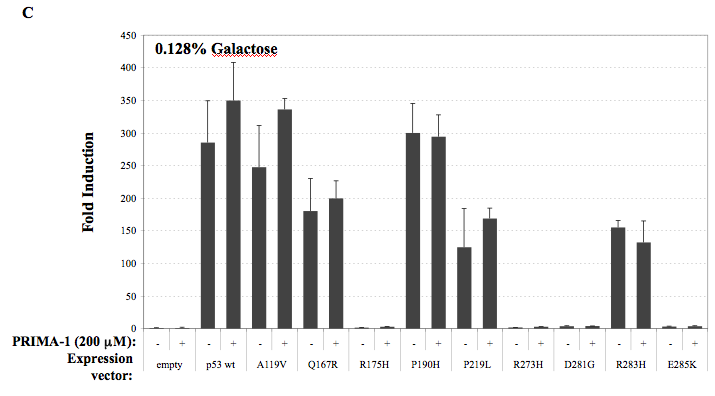

Supplement: Supporting Information S1 — 1. Small-volume yeast functional assay with constitutive expression of p53 proteins. Presented is the comparison of the relative transactivation capacity of wild type (WT) and the R282Q p53 towards four different response elements (REs) obtained with the traditional assay based on 2 ml liquid cultures in individual tubes (A, traditional assay) and with the permeabilized assay format based on 100 µl cultures prepared directly in 96-well plates (C, miniaturized assay). p53 proteins were expressed under the moderate, constitutive ADH1 promoter. Cells collected from the two different culture protocols were used for the measurement of luciferase activity as described in the Materials and Methods section. Presented are the average fold-induction of luciferase by p53 proteins relative to the activity obtained with an empty vector; included is the standard deviations of three replicates. In these experiments the light units per OD for WT p53 and the p21-5′ RE were 2.8×10^6^ for the 2 ml cultures and 2.5×10^7^ for the 100 µl cultures. 2. Impact of genetic modifications at the ABC transporter system on cell sensitivity to cycloheximide. Based on the experiments described by Stepanov et. al. [39] we used cycloheximide treatment to evaluate whether the disruption of PDR1 and replacement with the PDR1-repressor construct, the disruption of PDR5, or the combined modifications would result in enhanced toxicity in our reporter strain background. Cells from the indicated strains were resuspended in sterile water and transferred to a 96-well plate. Serial dilutions (1:5) were prepared and cells were transferred to plates containing synthetic medium (SD) with different concentrations of cycloheximide using a 48-pin replicator. A rich (YPDA) and an SD control plates were also spotted for comparison. Plates were incubated for two days at 30°C. 3. Phenotypic analysis of the impact of MDM2 on WT and mutant p53 transactivation. The ADE2-based red/white assay was used to examine p53 dependent t [file pone.0020643.s001.doc]
